# Transcriptome and Gene Expression Analysis of the Rice Leaf Folder, *Cnaphalocrosis medinalis*

**DOI:** 10.1371/journal.pone.0047401

**Published:** 2012-11-19

**Authors:** Shang-Wei Li, Hong Yang, Yue-Feng Liu, Qi-Rong Liao, Juan Du, Dao-Chao Jin

**Affiliations:** Provincial Key Laboratory for Agricultural Pest Management of the Mountainous Region, Institute of Entomology, Guizhou University, Guiyang, China; New Mexico State University, United States of America

## Abstract

**Background:**

The rice leaf folder (RLF), *Cnaphalocrocis medinalis* (Guenee) (Lepidoptera: Pyralidae), is one of the most destructive pests affecting rice in Asia. Although several studies have been performed on the ecological and physiological aspects of this species, the molecular mechanisms underlying its developmental regulation, behavior, and insecticide resistance remain largely unknown. Presently, there is a lack of genomic information for RLF; therefore, studies aimed at profiling the RLF transcriptome expression would provide a better understanding of its biological function at the molecular level.

**Principal Findings:**

*De novo* assembly of the RLF transcriptome was performed via the short read sequencing technology (Illumina). In a single run, we produced more than 23 million sequencing reads that were assembled into 44,941 unigenes (mean size = 474 bp) by Trinity. Through a similarity search, 25,281 (56.82%) unigenes matched known proteins in the NCBI Nr protein database. The transcriptome sequences were annotated with gene ontology (GO), cluster of orthologous groups of proteins (COG), and KEGG orthology (KO). Additionally, we profiled gene expression during RLF development using a tag-based digital gene expression (DGE) system. Five DGE libraries were constructed, and variations in gene expression were compared between collected samples: eggs vs. 3^rd^ instar larvae, 3^rd^ instar larvae vs. pupae, pupae vs. adults. The results demonstrated that thousands of genes were significantly differentially expressed during various developmental stages. A number of the differentially expressed genes were confirmed by quantitative real-time PCR (qRT-PCR).

**Conclusions:**

The RLF transcriptome and DGE data provide a comprehensive and global gene expression profile that would further promote our understanding of the molecular mechanisms underlying various biological characteristics, including development, elevated fecundity, flight, sex differentiation, olfactory behavior, and insecticide resistance in RLF. Therefore, these findings could help elucidate the intrinsic factors involved in the RLF-mediated destruction of rice and offer sustainable insect pest management.

## Introduction

The rice leaf folder (RLF), *Cnaphalocrocis medinalis* (Guenee) (Lepidoptera: Pyralidae), is an insect with migratory characteristics that poses a major pest threat to rice plants. RLF is widely found in rice-growing areas of Asia, Oceania, northeastern Australia, and Madagascar [1]. Outbreaks of serious RLF infestations have been reported in many Asian countries, including China, India, Pakistan, Japan, Korea, Malaysia, Sri Lanka, and Vietnam [2], [3]. The larvae of this species damage plants by feeding on leaves, resulting in patches of missing green leaf tissue. In China in 2011, this pest infested the rice-growing tracts of the middle-lower Yangtze area, south of the Yangtze River, South China, as well as the eastern part of Southwest China, damaging 22 million hm^2^ of rice. Since 2003, the occurrences of RLF infestation have been increasingly serious in China, and the annual average rice area infested by RLF was more than 20 million hm^2^, reducing the grain yield by up to 760 million kg every year.

RLF damages rice crops in its larval stage. The larva folds a leaf blade together, glues it longitudinally with silk strands, and feeds inside the rolled leaf, thereby creating longitudinal white and transparent streaks on the blade, which eventually withers. A single larva can damage a number of leaves, disturbing their photosynthesis and growth and ultimately reducing the yield [4]. To avoid yield loss, *C. medinalis* population is currently managed with chemical controls; however, the use of insecticides has failed because of pest-resistance, resulting environmental pollution, and pest-resurgence [5]. Therefore, it is imperative to determine an alternative approach to control this pest.

Extensive studies have been performed on the ecology, toxicology, behavior, and physiology of RLF, such as the population dynamics, insecticide evaluations, resistance monitoring, natural enemies, and long-distance displacements [4], [6], [7]. Several important factors favoring RLF development include changes in the physical environments, cultural practices, multiple rice cropping, reduced genetic variability of high yielding rice varieties, heavy use of fertilizer, and the misuse of pesticides [5], [8], [9]. The intrinsic RLF characteristics contribute to the insect pest population increase and the resulting catastrophic events. Therefore, it is necessary to determine the inherent mechanisms and characteristics of RLF. However, the regulatory molecular mechanisms in this species remain largely unknown.

Over the past several years, next-generation sequencing (NGS) techniques have been developed to generate large volumes of sequencing data inexpensively [10]. NGS affords us unprecedented high-throughput and low-cost sequencing platforms applied in a variety of manners, including *de novo* whole-genome sequencing, resequencing of genomes to identify variations, *de novo* transcriptome and gene expression profiling, and detecting methylation patterns [11], [12]. Currently, the Illumina/Solexa sequencing technology dominates the NGS market, featuring high data accuracy and a broad range of applications.

In this paper, we implemented *de novo* transcriptome assembly and digital gene expression (DGE) profiling analysis using Illumina sequencing technology. More than 23 million reads were generated and assembled into 44,941 unigenes of the RLF transcriptome. Furthermore, five DGE libraries were constructed for the purpose of comparing RLF gene expression profiles at different developmental phases. The obtained RLF transcriptome and DGE data provide essential information for identifying the genes involved in developmental regulation, flight activity, sex differences, olfactory behavior, and insecticide resistance.

## Methods

### Insect rearing and sample preparation

Adult RLFs were originally collected from a paddy field in the experimental farm of Guizhou University, which is located in Guiyang, China. The insects were reared in a climate chamber on rice plants (Jinyou 431) at 25±1°C under a 14∶10 light∶dark photoperiod and at 80% relative humidity. The offspring of a couple were used as experimental insects. Eggs were collected from the rice leaf blades, and 1^st^ to 5^th^ instar larvae were distinguished by body size and color difference. All insect samples were placed in RNAlater (Invitrogen, Carlsbad, CA) and stored at −80°C until use.

### RNA extraction and cDNA library preparation for transcriptome analysis

Total RNAs were extracted from RLF samples using the SV total RNA isolation system (Promega, Madison, WI) according to the manufacturer's protocol. RNA obtained from eggs, 1^st^ to 5^th^ instar larvae, pupae, and adults was merged into one sample at equal ratios for transcriptome analysis. Additionally, mRNA sequencing samples were prepared using the mRNA-seq sample preparation kit (Illumina, San Diego, CA). Following the Illumina manufacturer's procedures, mRNA was purified from 10 µg of the pooled total RNA using polyT oligo-attached magnetic beads. Fragmentation buffer was added to disrupt the mRNA into short fragments. Reverse transcriptase and random primers were used to synthesize the first strand cDNA from the cleaved mRNA fragments. The second strand cDNA was synthesized using buffer, dNTPs, RNase H, and DNA polymerase I. These cDNA fragments were purified with the QIAquick PCR purification kit (QIAGEN, Hilden, Germany) and subjected to the end repair process that added an ‘A’ base to the 3′ end of the cDNA. Next, the short fragments were connected with sequencing adapters. These ligation products were subjected to agarose gel electrophoresis and the suitable fragments were amplified by PCR to construct a cDNA library.

### Bioinformatics analysis of sequencing results

The cDNA library was sequenced using the Illumina HiSeq 2000 System. Sequencing-received raw image data were transformed by base calling into sequence data, which were called raw reads. The clean reads, obtained after filtering dirty raw reads, were used for bioinformatics analysis. Transcriptome *de novo* assembly was performed separately with the short reads assembling programs SOAPdenovo [13] and Trinity [14]. It has been demonstrated that Trinity is a more efficient *de novo* transcriptome assembler, especially in the absence of a reference genome [14]. First, Trinity combined the reads with a certain overlap length to form longer fragments, which were called contigs. Next, these reads were mapped back to contigs; with paired-end reads, Trinity was able to detect contigs from the same transcript and determine the distances between these contigs. Finally, Trinity connected these contigs into sequences that could not be extended on either end. Such sequences were defined as unigenes. After clustering, the unigenes would be divided into two classes: clusters and singletons. The distinct unigenes were used for BLASTx searches and annotations with a cut-off E-value of 10^−5^ against protein databases, such as Nr, Swiss-Prot, KEGG, and COG. If the alignment results of different databases conflicted with each other, we followed the priority order of Nr, Swiss-Prot, KEGG, and COG when determining the unigene sequence direction. Gene Ontology (GO, www.geneontology.org) functional annotation of unigenes was performed by Blast2go software [15]. COG and KEGG annotations were analyzed by the Blastall program against the Clusters of Orthologous Groups of proteins (COG, www.ncbi.nlm.gov/COG) and Kyoto Encyclopedia of Genes and Genomes (KEGG, www.genome.jp/kegg) databases.

### Gene expression profile sequencing

Samples for RNA isolation included RLF eggs, 3^rd^ instar larvae, pupae, and adults (females and males). Total RNA from these samples was extracted for digital gene expression (DGE) libraries that were prepared by the Illumina gene expression sample prep kit (Illumina, San Diego, CA). Six micrograms of total RNA was used to purify mRNA with Oligo(dT) magnetic beads adsorption. The first and second cDNAs were synthesized and the bead-bound cDNAs were subsequently digested by *Nla* III, which cut cDNAs at the CATG sites. The digested 3′ cDNA fragments were connected to the Illumina adapter 1 at the sticky 5′ ends. The junction of adapter 1 and CATG site was the recognition site of *Mme* I, which cut the cDNA at a position 17 bp downstream of the CATG site, thereby producing tags with adapter 1. After removing 3′ fragments with magnetic beads precipitation, Illumina adapter 2 was ligated to the tags at the 3′ ends, acquiring tags with different adapters at both ends and thus generating a tag library. After 15 cycles of linear PCR amplification, 95 bp bands were purified by 6% PAGE gel electrophoresis. The single-stranded molecules were fixed onto the Illumina sequencing chip (flow cell) for sequencing by synthesis. Each tunnel generated millions of raw 49 bp reads.

### Bioinformatics analysis of digital gene expression (DGE) tags

To map the tags to the transcriptome database, the obtained raw sequence data were filtered by data processing steps to generate clean tags by a process that included the removal of adapter sequences, empty reads (reads with only adapter sequences but no tags), low-quality sequences (tags with unknown sequences ‘N’), and tags with a copy number of 1 (likely sequencing errors). A reference tag library containing all of the sequences of CATG plus 17 bases was created by searching the CATG sites in the transcriptome database. All clean tags were mapped to the reference library, permitting no more than a one-base mismatch. Clean tags, which were mapped to exactly one gene in the reference database, were designated as unambiguous tags for gene annotation. The number of unambiguous tags for each gene was calculated and normalized to TPM (number of transcripts per million tags) for the gene expression analysis [16], [17].

### Screening and analysis of differentially expressed genes

Implementing the method described by Audic and Claverie [18], a rigorous algorithm was developed to identify differentially expressed genes (DEGs) between two different DGE libraries. The false discovery rate (FDR) method determines the P-value threshold for multiple testing by controlling the FDR value. The criteria of FDR≤0.001 and the absolute value of log2ratio≥1 were used to judge the significance of gene expression differences. For GO enrichment analysis of functional significance, we applied the hypergeometric test to map all the differentially expressed genes to terms in the GO database, identifying GO terms significantly enriched for DEGs compared to the genome background. The calculating formula is
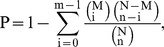
where N is the number of all genes with GO annotation, n is the number of DEGs in N, M is the number of all genes that are annotated to the certain GO terms, and m is the number of DEGs in M. A corrected P-value≤0.05 was set as a threshold to identify the significant enrichment of GO terms in differential gene expression. The differentially expressed genes were also utilized in KEGG ontology (KO) enrichment analyses to further understand their biological functions. Pathway enrichment analysis could identify significantly enriched metabolic pathways or signal transduction pathways in DGEs compared with the genome background. Pathways with Q-value≤0.05 were viewed as significantly enriched in DEGs.

### Validation of gene expression by quantitative real-time PCR

Quantitative real-time reverse transcription PCR (qRT-PCR) was performed to validate the mRNA sequencing data. Total RNA was extracted for the DGE library preparation as described above. One microgram of total RNA was reverse transcribed into single-stranded cDNA using the Primescript RT reagent kit (TaKaRa, Dalian, China). qRT-PCR was implemented using the SYBR premix Ex Taq kit (TaKaRa, Dalian, China), with the first strand cDNA serving as the template. The *α-tubulin* gene from RLF was utilized as an internal control. The relative quantitative method (ΔΔC_T_) was used to calculate the fold change of target genes [19]. The primers employed in the qRT-PCR are listed in Table S1.

## Results

### Illumina sequencing and reads assembly

A total of 23,865,844 reads (consisting of 2,147,925,960 bp) were produced by Illumina sequencing after cleaning and quality checking. We obtained 78,747 unigenes with a mean size of 332 bp using SOAPdenovo assembly. The results obtained by Trinity showed that these reads were assembled into 111,873 contigs via short overlaps. The mean contig size was 253 bp with lengths ranging from 200 bp to more than 3,000 bp. These contigs were further assembled into 44,941 unigenes with a mean length of 474 bp, which included 5,919 distinct clusters and 39,022 distinct singletons (Fig. S1). Evidently, Trinity performed much more effectively than SOAPdenovo in assembly quality; therefore, Trinity-assembled transcripts were used for downstream analysis. To evaluate the quality of sequencing and assembly, we chose 100 unigenes at random and designed RT-PCR amplification primers specific to these sequences. These PCR products were sequenced by the traditional Sanger method; the results demonstrated a 98% identity match, suggesting that the assembled unigenes were highly accurate. These reads were submitted to SRA at NCBI under the accession no. SRA058899.

### Annotation of predicted proteins

**Figure 1:**
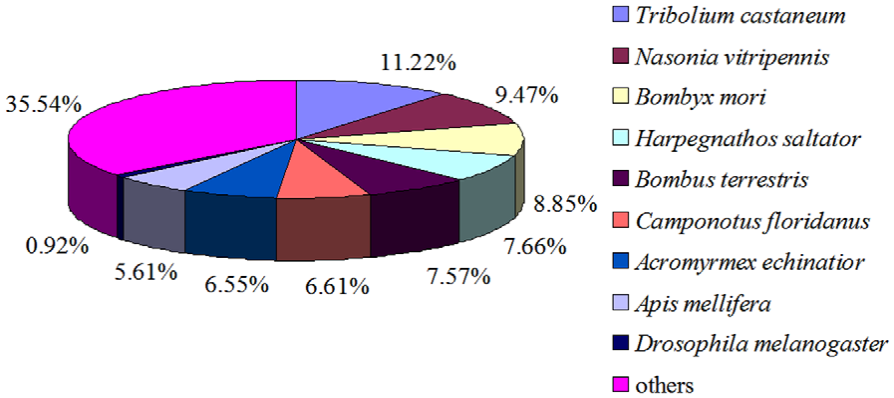
Species distribution of homology search of unigenes against the Nr database. The species distribution is shown as a percentage of the total homologous sequences in the NCBI Nr protein database with an E-value<10^−5^.

To annotate these unigenes, distinct sequences were first searched by BLASTx against the NCBI non-redundant protein database (Nr) with a cut-off E-value of 10^−5^. In total, 25,281 unigenes (56.82% of all distinct sequences) matched known genes (Table S2); the other 19,210 unigenes (43.18%) failed to acquire annotation information in the Nr database. The species distribution of the top BLAST hits for each unique sequence is shown in Fig. 1. The unambiguous unigenes from RLF revealed that the greatest number of matches (11.22%) was with sequences from the coleopterous species red flour beetle (*Tribolium castaneum*) followed by the sequences from the jewel wasp (*Nasonia vitripennis*) (9.47%), silkworm (*Bombyx mori*) (8.85%), and ponerine ant (*Harpegnathos saltator*) (7.66%). Additionally, the unigenes matched 43 of total 67 sequences from *C. medinalis* deposited in the Nr database.

### Unigene functional annotation by GO, COG, and KEGG

**Figure 2:**
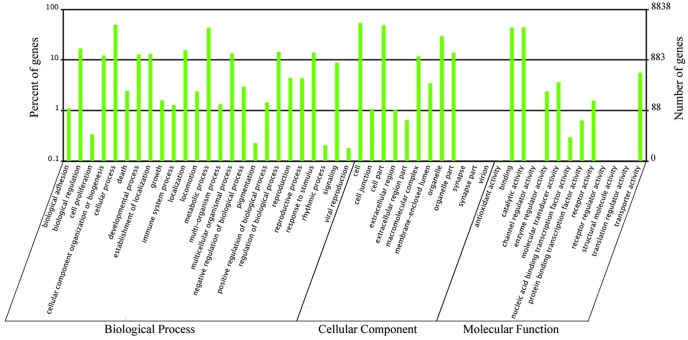
Gene ontology classification of the unigenes. A total of 8,838 unigenes were annotated by three categories: biological process, cellular component, and molecular function. The left and right y-axes denote separately the percent and number of genes in the category.

Gene Ontology (GO) is an international standardized gene functional classification system and covers three domains: cellular component, molecular function, and biological process. We used WEGO software to do GO functional classification for all the GO-annotated unigenes (Table S3) to understand the distribution of gene functions of this species at the macro level. The 8,838 unigenes (35% of total) were categorized into 50 functional groups. Cell and cellular process were the two largest groups, containing 4,747 and 4,378 unigenes, respectively. Synapse part and receptor regulator activity were the two smallest groups; each only comprised one unigene (Fig. 2).

**Figure 3:**
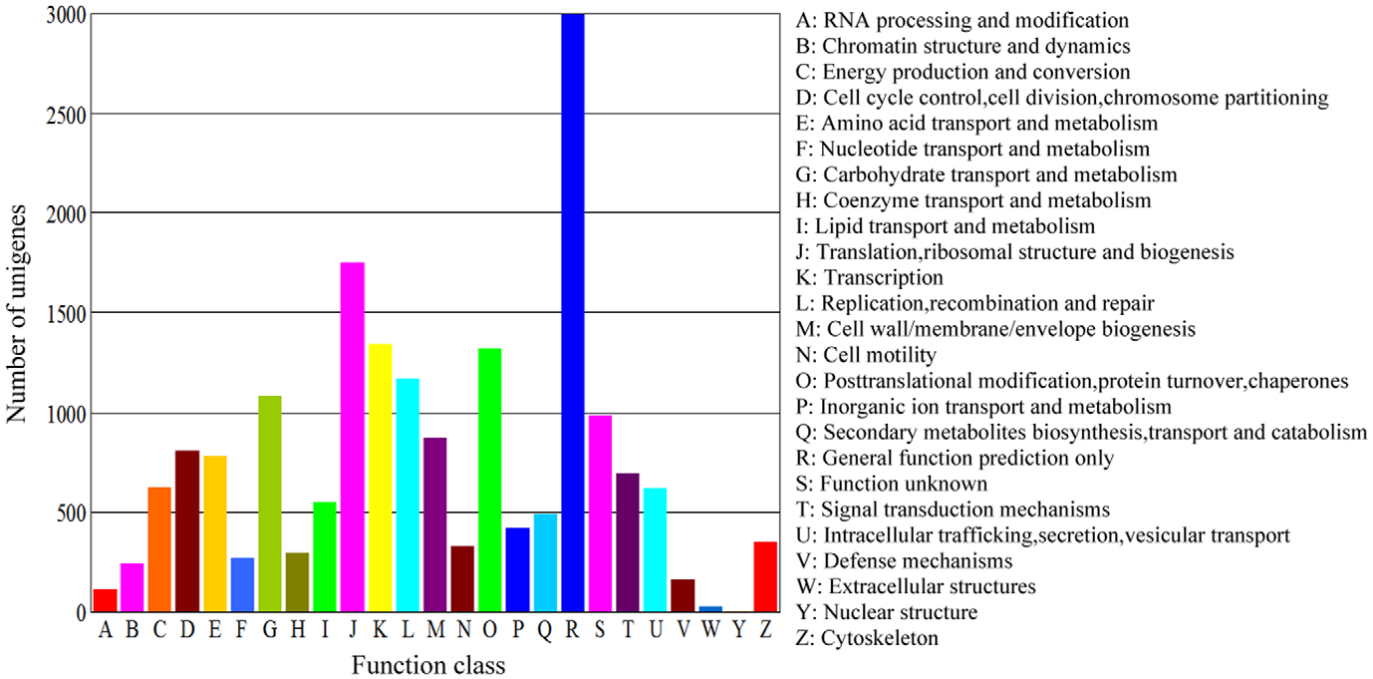
Clusters of orthologous groups (COG) classification of the unigenes. Out of 25,281 unigenes, 18,285 sequences fell into 25 categories in COG.

To more accurately annotate their functions, we aligned the unigenes to the COG database to find homologous genes. In total, 18,285 unigenes (72.33%) were annotated and formed 25 COG classifications (Fig. 3 and Table S4). Among the functional classes, the “general function prediction” cluster constituted the largest group (2,994, 16.37%) followed by “translation, ribosomal structure and biogenesis” (1,750, 9.57%) and “transcription” (1,342, 7.34%); the two smallest groups were “extracellular structures” (25, 0.137%) and “nuclear structure” (8, 0.044%).

To identify the metabolic pathways populated by these unigenes, the 25,281 annotated sequences were mapped to the Kyoto Encyclopedia of Genes and Genomes (KEGG) pathway database. A total of 17,705 sequences were annotated to 242 KEGG pathways. The pathways with the most unigenes were “metabolic pathways” (3096, 17.49%); “polyketide sugar unit biosynthesis” (2, 0.01%) and “intestinal immune network for IgA production” (2, 0.01%) pathways were populated by the fewest number of unigenes. These annotations laid the groundwork for further research of metabolic pathways, functions, and complicated biological behaviors of RLF genes.

### Transcripts encoding the insecticide detoxification and target enzymes

Insecticide resistance represented a specific concern; therefore, we detected the unigenes related to metabolic resistance induced by increasing the metabolic capabilities of detoxifying enzymes and target site resistance. Generally, cytochrome P450 (P450), carboxylesterase (CarE), and glutathione S-transferase (GST) are the three primary enzymes involved in the detoxification of insecticides. Many transfrags encoding insecticidal targets were identified, including acetylcholinesterase (AChE) (unigene7280), γ-aminobutyric acid receptor (GABA) (unigene33609), nicotinic acetylcholine receptor (nAChR) (unigene33738), and sodium channel (unigene6017). Specific sequence information for these unigenes is shown in Table S5.

**Figure 4:**
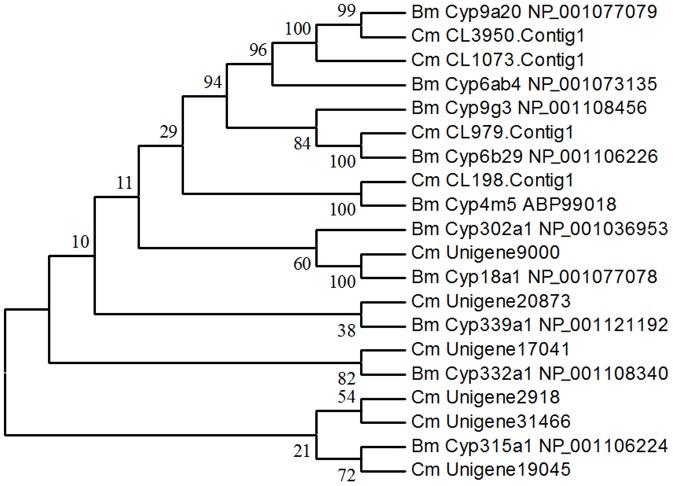
Phylogenetic tree of cytochrome P450s from *Cnaphalocrocis medinalis* (Cm) and *Bombyx mori* (Bm). The tree was constructed from the multiple alignments using MEGA 5.0 software and generated with 1,000 bootstrap trials using the neighbor-joining method. The numbers indicate the bootstrap confidence values obtained for each node after 1,000 repetitions.

**Figure 5:**
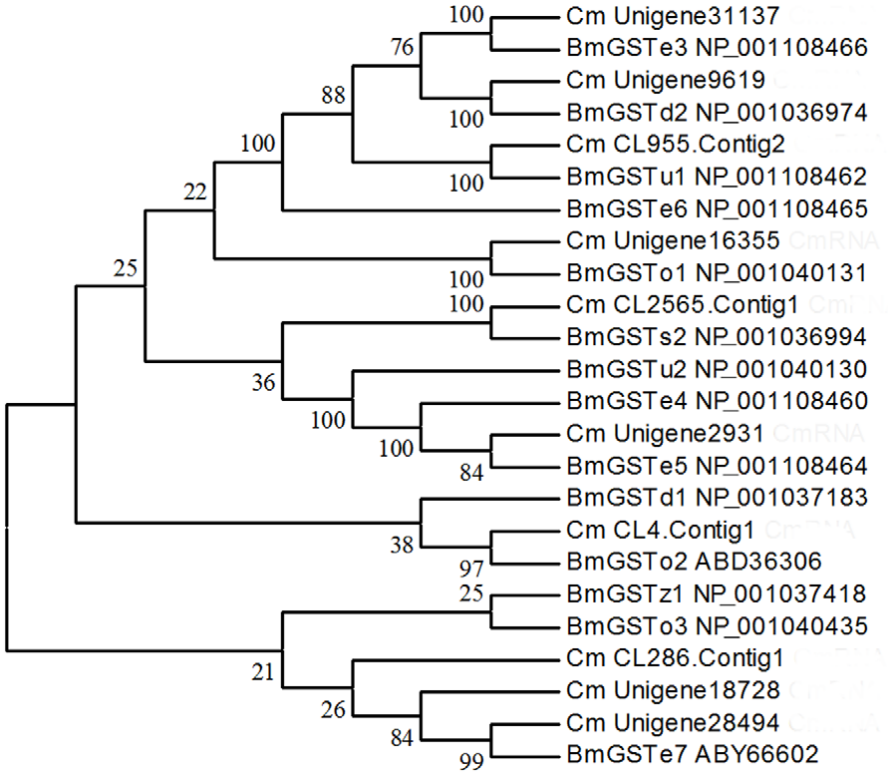
Phylogenetic tree of glutathione S-transferases from *Cnaphalocrocis medinalis* (Cm) and *Bombyx mori* (Bm). The tree was constructed from the multiple alignments using MEGA 5.0 software and generated with 1,000 bootstrap trials using the neighbor-joining method. The numbers indicate the bootstrap confidence values obtained for each node after 1,000 repetitions.

**Figure 6:**
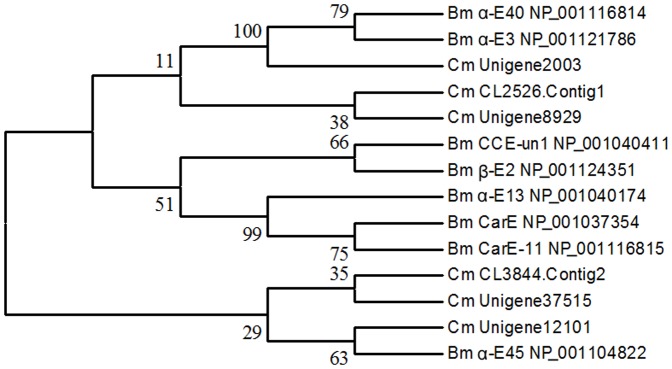
Phylogenetic tree of carboxylesterases from *Cnaphalocrocis medinalis* (Cm) and *Bombyx mori* (Bm). The tree was constructed from the multiple alignments using MEGA 5.0 software and generated with 1,000 bootstrap trials using the neighbor-joining method. The numbers indicate the bootstrap confidence values obtained for each node after 1,000 repetitions.

A total of 91 cytochrome P450-related transfrags were found in our transcriptome data, of which 10 sequences averaging 1,366 bp in length were identified as P450-specific genes. Based on the best BLAST matches against the Nr database and phylogenetic analysis with P450 genes from *B. mori*, they belonged to *CYP4*, *CYP6*, *CYP9*, *CYP18A*, *CYP301*-*CYP318*, and *CYP332*-*CYP343* families and subfamilies (Fig. 4). Among the 48 GST-related transfrags in the transcriptome, 10 sequences with an average length of 978 bp were identified by comparing with GST genes from *B. mori*. Phylogenetic analysis showed that these genes were classified as delta, epsilon, zeta, sigma, omega, and an unclassified class (Fig. 5). Six sequences with an average length of 1,201 bp were identified as CarE genes from 158 transfrags of esterases. Phylogenetic comparison with genes from *B. mori* indicated that these sequences had homology with α-esterases (Fig. 6).

### Phylogenetic analysis of the RLF OBP genes

**Figure 7:**
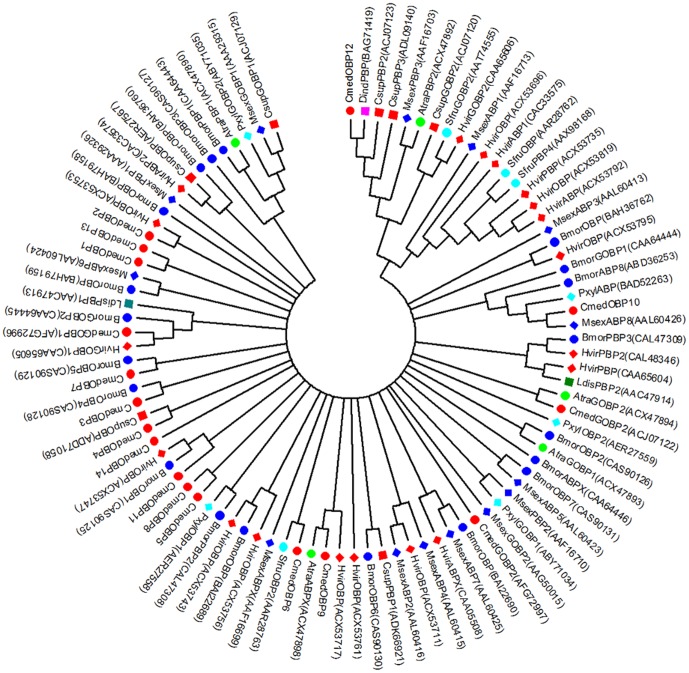
An unrooted consensus tree of annotated OBP proteins among *C. medinalis* and other Lepidopteran insects. This tree was constructed from the multiple alignments using MEGA 5.0 software, generated with 1,000 bootstrap trials using the neighbor-joining method, and presented with a cut-off value of 10%. For most OBPs, names are presented as a three-letter code (first letter of genus followed by first two letters of species name) followed by the GenBank accession number in the parenthesis at the end of each branch. The insect species are indicated by colored markers. Atra, *Amyelois transitella*; Bmor, *Bombyx mori*; Cmed, *Cnaphalocrocis medinalis*; Csup, *Chilo suppressalis*; Dind, *Diaphania indica*; Hvir, *Heliothis virescens*; Ldis, *Lymantria dispar*; Msex, *Manduca sexta*; Pxyl, *Plutella xylostella*; Sfru, *Spodoptera frugiperda*.

Odorant-binding proteins (OBPs) are a class of small, water-soluble, extracellular proteins that are thought to aid in the capture and transport of odorants and pheromones to olfactory receptors (ORs). In total, 25 OBP-related sequences were obtained from our transcriptome analysis. Based on both the best match in the Nr database and the phylogenetic analysis with other lepidopteran OBPs, 14 sequences with an average length of 561 bp were identified as OBP genes (CmedOBP1-14). Among these sequences, eight (Cmed1-8) belonged to general odorant-binding proteins (GOBPs), three (Cmed9-11) belonged to antennal-binding proteins (ABPs), and the other three (Cmed12-14) belonged to pheromone-binding proteins (PBPs) (Fig. 7 and Table S6).

### Digital gene expression (DGE) library sequencing

To measure the absolute mRNA expression levels during RLF development, gene expression variations were analyzed by the DGE approach. Five DGE libraries from RLF were sequenced: eggs, 3^rd^ instar larvae, pupae, male adults, and female adults, each of which produced approximately six million raw tags. After filtering low quality tags from the raw data, the total number of clean tags per library varied from 5.75 million to 5.99 million, with the corresponding percentage among the raw tags ranging from 97.21% to 98.41% (Fig. S2). The number of clean tags that could be mapped to the reference unigenes varied from 3.09 million to 3.92 million, with the corresponding percentage in all clean tags ranging from 53.78% to 65.29% in the five libraries (Table 1). Generally, heterogeneity and redundancy are the two significant characteristics of mRNA expression. Only a small number of mRNAs have high abundance, while the majority of mRNAs maintain a low level of expression. Thus, the distribution of clean tag expression could be used to evaluate the normality of the DGE data. As shown in Fig. S3, at each developmental stage, the highly expressed genes with more than 100 copies accounted for more than 80% of the clean tags, whereas their tag categories did not exceed 8%. Conversely, the low-level expressed genes with fewer than 5 copies occupied more tag categories than represented by 54% of the distinct clean tags (Fig. S3). These reads were submitted to SRA at NCBI under the accession no. SRA058899.

**Table 1:** Statistics of DGE sequencing.

Summary	Eggs	3^rd^ instar larvae	Pupae	Male Adults	Female Adults	Averages
Raw Data	6,115,004	6,066,042	5,961,614	6,126,227	5,911,019	6,035,981
Distinct Raw Data	206,746	232,570	168,371	220,376	264,404	218,493
Clean Tag	5,998,487	5,944,052	5,867,100	5,988,301	5,746,384	5,908,865
Distinct Clean Tag	90,360	110,650	73,963	90,975	106,094	94,408
Clean Tag/Raw Tag	98.09%	97.99%	98.41%	97.75%	97.21%	97.89%
All Tag Mapping to Gene	3,916,598	3,475,226	3,698,670	3,310,426	3,090,658	3,498,316
All Tag Mapping to Gene1)	65.29%	58.47%	63.04%	55.28%	53.78%	59.17%
Distinct All Tag Mapping to Gene	34,495	35,118	27,988	29,216	35,241	32,412
Distinct All Tag Mapping to Gene1)	38.18%	31.74%	37.84%	32.11%	33.22%	34.62%
Unambiguous Tag mapping to Gene	3,554,919	3,276,273	3,340,732	2,822,317	2,788,593	3,156,567
Unambiguous Tag mapping to Gene1)	59.26%	55.12%	56.94%	47.13%	48.53%	53.40%
Distinct Unambiguous Tag mapping to Gene	30,788	32,362	24,948	25,748	31,796	29,128
Distinct Unambiguous Tag mapping to Gene1)	34.07%	29.25%	33.73%	28.30%	29.97%	31.06%
All Tag-mapped Genes	13,133	14,591	11,075	11,287	13,147	12,647
All Tag-mapped Genes2)	29.22%	32.47%	24.64%	25.12%	29.25%	28.14%
Unambiguous Tag-mapped Genes	11,384	12,885	9,493	9,693	11,484	10,988
Unambiguous Tag-mapped Genes2)	25.33%	28.67%	21.12%	21.57%	25.55%	24.45%
Unknown Tag	1,770,338	2,193,724	1,883,472	2,470,385	2,302,386	2,124,061
Unknown Tag1)	29.51%	36.91%	32.10%	41.25%	40.07%	35.97%
Distinct Unknown Tag	51,283	69,065	42,302	57,548	65,191	57,078
Distinct Unknown Tag1)	56.75%	62.42%	57.19%	63.26%	61.45%	60.21%

Notes:

1) , % of Clean Tag;

2) , % of Reference Unigenes.

### Gene expression differences among the various developmental phases

**Figure 8:**
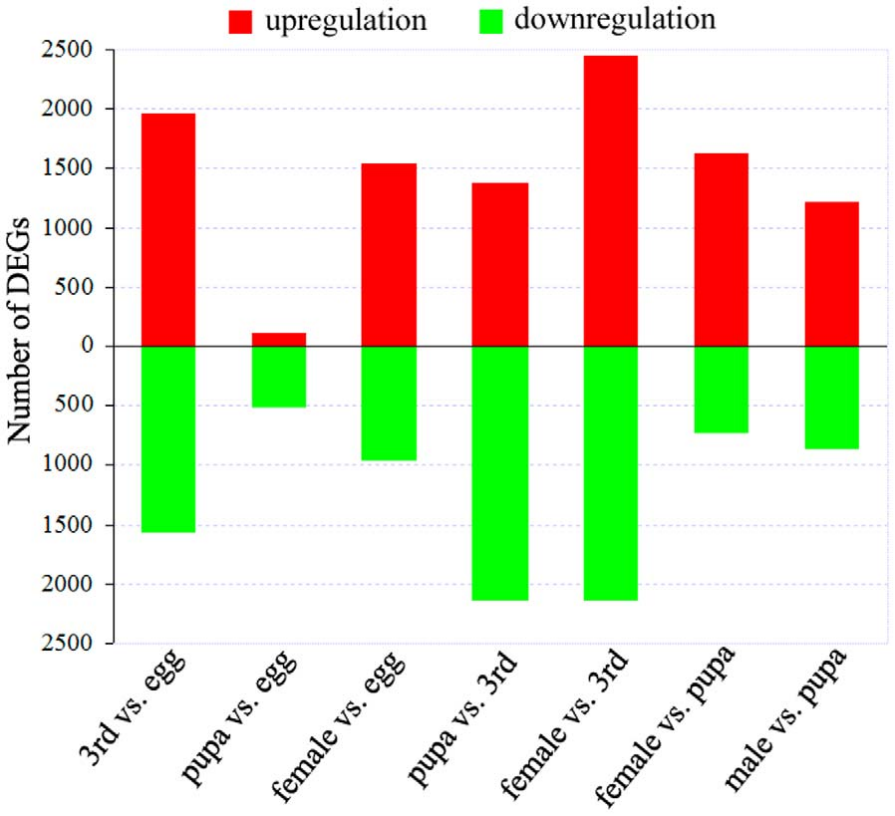
Differentially expressed genes (DEGs) between the different developmental phases of RLF. This figure shows the number of upregulated (red) and downregulated (green) genes in each pairwise comparison of eggs, 3^rd^ instar larvae, pupae, and adults.

To identity genes showing significant changes in expression levels during different developmental phases, the differentially expressed tags between the two specimens were identified by a rigorous algorithm [18]. Changes in the gene-expression profiles were analyzed by comparisons between eggs and 3^rd^ instar larvae, 3^rd^ instar larvae and pupae, and pupae and adults. A total of 3,535 genes were significantly differentially expressed between the eggs and 3^rd^ instar larvae libraries. Of these genes, 1,960 were upregulated and 1,575 were downregulated in the 3^rd^ instar larvae compared with the eggs (Fig. 8). In the 10 most upregulated genes, only 3 out of 10 had defined functions: *juvenile hormone epoxide hydrolase*, *60S ribosomal protein L7*, and *hemolymph protein*. In the 10 most downregulated genes, 3 had specific functional annotations: *cuticular protein RR-1 motif 37* (*CPR37*), *β-fructofuranosidase 2*, and *chorion protein s18* (Table S6). Based on the GO classification, most of the upregulated gene sets in the 3^rd^ instar larva stages were associated with metabolic processes, implying that metabolic activities in the larvae were stronger than in the eggs. Additionally, the gene sets enriched in KO were chiefly involved in metabolic pathways (Table S8).

Comparing pupae with 3^rd^ instar larvae, there were 3,525 transcripts with remarkable expression variations in the pupa stage, including 1,385 upregulated and 2,140 downregulated genes. An analysis of the 10 most differentially upregulated and 10 downregulated genes revealed a total of 17 genes with homologues found in the NCBI Nr database. Among these upregulated genes, three genes pertained to cuticular proteins; the other three matched the genes encoding peritrophin type-A domain protein 2, β-fructofuranosidase 2, and chorion protein s18. Four of the five downregulated genes encoded ribosomal proteins, while the remaining gene was *elongation factor 1-alpha* (*EF-1α*). According to the GO sorting, most of the gene sets that displayed downregulated expression in the pupa stage were primarily related to intracellular components (Table S9). Most of the gene sets significantly enriched in this comparison were correlated with metabolic pathways based on the KO sorting (Table S9).

Between the adult and pupa libraries, a total of 2,361 differentially expressed genes were detected in the female, with 1,635 upregulated and 726 downregulated genes; in the male, there were 2,096 differential genes, comprising 1,219 upregulated and 877 downregulated genes (Fig. 8 and Table S7). Examining the 20 most differentially expressed genes between the female adult and pupa stages, seven genes had defined functional annotations. Among the top 10 upregulated genes, four were identified as histone 1, follicular epithelium yolk protein sybunit, peritrophic matrix protein, and vitellogenin receptor. Three of the top 10 downregulated genes were functionally defined as storage protein, α-crystallin, and cuticular protein. In the top 20 differentially expressed genes between the male adult and pupa stages, 13 genes had defined annotations. Two of the five upregulated genes were *seminal fluid proteins*, and the other three genes were *glue silk protein* (*PySp1*), *PFTAIRE-interacting factor 2* (*Pif2*), and *cAMP-dependent protein kinase*. Six of the eight downregulated genes were *cuticular proteins*; the other two genes were *storage proteins*. Most of the gene sets enriched in GO were upregulated in the adult stage and involved in metabolic processes, e.g., macromolecule metabolic processes and in intracellular components (Table S10). Among the KO gene set enrichment, most genes also participated in metabolic pathways (Table S10).

### Functional annotation of differentially expressed genes

Different genes usually cooperate with each other to exercise their biological functions. To comprehend the functions of the differentially expressed genes, all of the genes were mapped to the KEGG ontology (KO) terms to identify genes involved in metabolic or signaling pathways. There were a total of 17,705 genes with KEGG pathway annotation; however, 1,753 differentially expressed genes with pathway annotations were identified between the egg and 3^rd^ instar larva stages. Evidently, specific gene enrichment focused on metabolism (24.99%), oxidative phosphorylation (6.56%), and ribosome (5.88%) pathways. Similarly, between the 3^rd^ instar larva and pupa stages, 1,753 differentially expressed genes with annotation were identified, and the primarily enriched pathways were involved with metabolism (25.33%), oxidative phosphorylation (6.62%), and ribosome (5.76%). A total of 811 differential genes with pathway annotation were identified between the pupa and adult stages. The specific gene enrichment was primarily observed for pathways involved in metabolism (21.76%), ribosome (7.01%), and RNA transport (6.46%). These results clearly demonstrated that the genes involved in metabolic pathways revealed significant changes in gene expression during RLF development.

### Gene expression analysis and qRT-PCR validation

**Figure 9:**
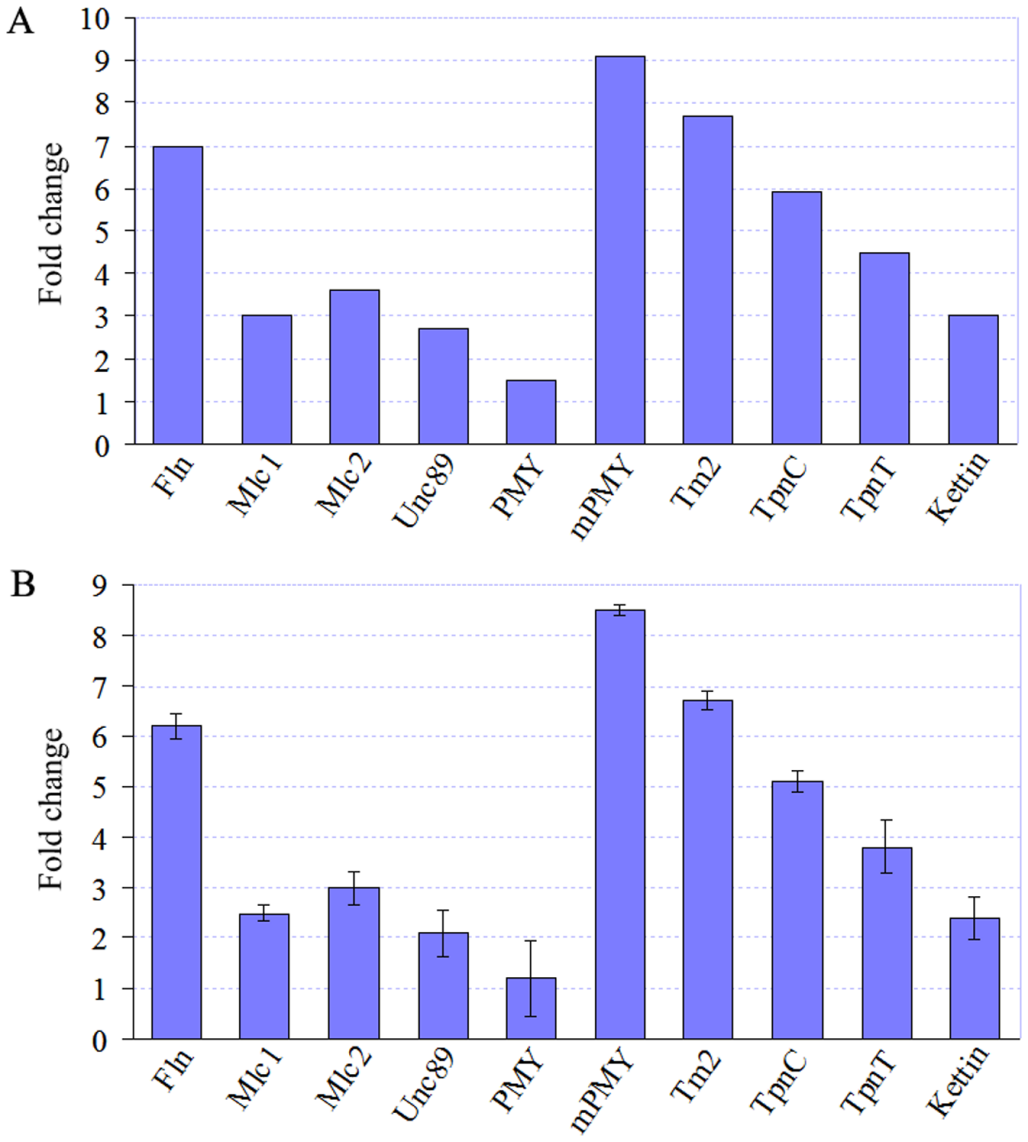
Genes in flight muscles from adult RLF. A: Gene expression analysis from DGE data. The fold changes in gene expression were calculated as the log2 ratio of adults (TPM) to 3^rd^ instar larvae (TPM). B: qRT-PCR analysis of gene expression. This figure shows the fold change of differential expression for 10 genes (*Fln*, *Mlc1*, *Mlc2*, *Unc89*, *PMY*, *mPMY*, *Tm2*, *TpnC*, *TpnT*, *Tm1*, and *Kettin*) in adults compared with 3^rd^ instar larvae.

We also examined the genes that play important roles in determining flight ability in the flight muscles of RLF. The genes pertinent to muscle cytoskeletal proteins had much higher expression levels in adults compared to 3^rd^ instar larvae. These genes included *flightin* (*Fln*), *myosin light chain alkali* (*Mlc1*), *myosin regulatory light chain 2* (*Mlc2*), *paramyosin*, *miniparamyosin*, *tropomyosin* (*Tm2*), *troponin* (*TpnC and TpnI*), and *kettin* (Fig. 9A and Table S11). Moreover, qRT-PCR results for these genes confirmed observations from DGE sequencing (Fig. 9B).

**Figure 10:**
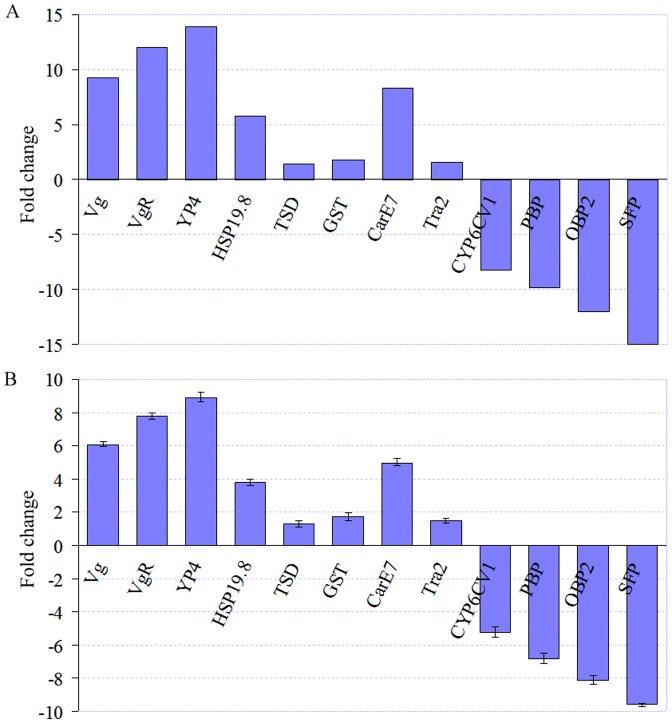
Genes related to sex differences. A: Gene expression analysis from DGE data. The fold changes in gene expression were calculated as the log2 ratio of females (TPM) to males (TPM). B: qRT-PCR analysis of gene expression. This figure shows the fold change of differential expression for 12 genes (*Vg*, *VgR*, *YP4*, *HSP19.8*, *TSD*, *GST*, *CarE7*, *Tra2*, *CYP6CV1*, *PBP*, *OBP2*, and *SFP*) in females compared with male adults.

By comparing the female and male adults, yolk protein genes, including *vitellogenin* (*Vg*), *vitellogenin* receptor (*VgR*), and *follicular epithelium yolk protein subunit* (*YP4*), were expressed exclusively in females, whereas *seminal fluid protein* (*SFP*) was expressed exclusively in males. Additionally, three sex difference-related genes, including *small heat shock protein 19.8* (*HSP19.8*), *transformer 2* (*Tra2*), and *temperature-dependent sex determining protein* (*TSD*) manifested upregulated expression levels in females compared to males. Moreover, *glutathione S-transferase* (*GST*) and *Carboxylesterase* (*CarE7*) were expressed at higher levels in females than in males. Furthermore, several other genes relevant to sex differences showed higher expression levels in males, such as *pheromone binding protein* (*PBP*), *odorant binding protein* (*OBP2*), and *cytochrome P450* (*CYP9CV1*) (Fig. 10A and Table S12). All of these genes were amplified by qRT-PCR, and the results were consistent with those acquired by DGE profiling (Fig. 10B).

## Discussion

The rice leaf folder *C. medinalis* has become a major threat to rice production in many Asian countries. The inherent molecular structure of RLF determines its characteristics, such as leaf folding, prolificacy, and dispersal. Therefore, an exhaustive understanding of molecular mechanisms regulating RLF behavior and activities may allow us to control the migratory pest with more sustainable and environmentally friendly approaches.

Surprisingly, BLASTx annotation of RLF transcriptome sequences revealed the highest similarity with *T. castaneum* of the order Coleoptera, while demonstrating a lower identity match (8.85%) with *B. mori*, a representative of the order Lepidoptera, which also encompasses RLF. To explain this similarity, we determined the number of protein sequences from *T. castaneum*, *N. vitripennis*, and *B. mori* in the Nr database and found 27,273, 10,637, and 6,842 protein sequences, respectively. It is clear that the protein sequences available in the database are fewer for *B. mori* than for *T. castaneum*. Therefore, the results may be due to the fewer protein sequences available for *B. mori*; however, the relationship between these species requires further investigation.

To the best of our knowledge, our work represents the first analysis of the RLF transcriptome and DGE profile data that greatly complemented and enriched the RLF database potentially to facilitate the determination of novel genes, gene functional analysis, insecticidal targets, developmental molecular mechanisms, and sex differentiation of RLF. Five DGE libraries of various developmental stages were constructed to analyze RLF gene expression patterns. In the RLF holometabolous development, the number of differentially expressed genes between the pupa and the adult was smaller than that between the pupa and the 3^rd^ instar larva; the latter was almost as large as that between the 3^rd^ instar larva and the egg. When examining the 3^rd^ instar larva differential gene expression, the number of upregulated genes was slightly more than the downregulated genes compared to the egg. The majority of upregulated genes were involved in metabolic processes, suggesting that the 3^rd^ instar larvae have stronger metabolic activities. In the pupal stage, the number of downregulated genes was greater than the upregulated genes compared with the 3^rd^ instar larvae. There were fewer differentially expressed genes in the pupa compared to the adult and more differentially expressed genes in the pupa compared to the larva. This result most likely indicates that pupa development occurs at the time of histogenesis when the old tissues have been destroyed and the new adult tissues or organs are being formed. Each RLF developmental stage demonstrates a large number of specifically differentially expressed genes that are most likely associated with developmental differentiation (Table S13). Insect metamorphosis and development are physiological behaviors that are modulated by the procedural and tissue-specific expression of a series of genes under hormonal control. Several genes, such as *EcR* (*ecdysone receptor*), *USP* (*ultraspiracle*), *JHBP* (*juvenile hormone binding protein*), *Kr-h1* (Kruppel homolog 1), and *broad*, were detected in the transcriptome database (Table S2); it is believed that these genes play key roles in holometabolous development of insects [20], [21].

In China, RLF has been primarily controlled by chemical methods over the years; therefore, under long-term selection pressures, the insect has developed resistance to commonly used insecticides, such as chlorpyrifos, trichlorphon, and carbosulfan. However, little is known about the molecular mechanisms behind the resistance of RLF. Our transcriptome data provide an enormous amount of genetic information that would facilitate research on resistance monitoring, mechanisms, and management strategy of the pest. Insect resistance to insecticides typically involves increases in the metabolic capability of detoxificative enzymes, as well as decreases in target site sensitivity; the former could be a more flexible way for insects to cope with xenobiotics. P450s and their associated P450 reductases can mediate resistance to all classes of insecticides via the upregulation and point gene mutations [22]. In this study, we have characterized an additional 10 RLF P450 genes that fall into six families. Another important group of metabolic enzymes are GSTs that can mediate resistance to organophosphate, organochlorines, and pyrethroids through gene amplification and overexpression [23]. To date, none of the GST genes from RLF has been reported in GenBank. We identified 10 GST genes of RLF and grouped them into six classes, thus filling the research gap. CarEs are also a class of important metabolic enzymes that are implicated in the resistance of insects to organophosphate, carbamates, and pyrethroids through gene amplification, upregulation, and coding sequence mutations [22], [24], [25]. Six CarE genes were identified in the RLF transcriptome, thus providing new genetic resources where previously no such gene information existed. In view of this finding, our research provides the foundation for understanding the molecular mechanisms underlying insecticide resistance and further implementation of environmentally sustainable pest management.

The insect olfactory system is a highly specific and sensitive chemical detector essential for feeding, mating, finding sites of oviposition and development, and withdrawing from hostile environments. In insects, the recognition of odorants occurs through a complex series of events that involve a variety of proteins, such as OBP, CSP (chemosensory protein), and OR (odorant receptor). Hydrophobic odorant molecules are combined with OBPs in the hydrophilic lymph and are transported to reach ORs on the olfactory neuron twig, leading to activation. This result evokes olfactory nerve excitation that passes to the nerve center, leading to the perception of odorants [26], [27], [28]. It has been believed that OBPs have key functions in recognizing and delivering hydrophobic odorants to ORs. We identified 14 OBPs from *C. medinalis*, which were classified into three groups: GOBP, PBP, and ABP. Additionally, 8 CSPs (e. g. unigene846 and unigene2263) were found in the RLF transcriptome data and were believed to perform similar roles with OBPs, despite their different structures [29]. These data provide valuable new information for further research on molecular mechanisms of RLF olfactory behavior.

*C. medinalis* is a migratory rice pest that possesses sufficient flight capacity for dispersal. It is thought that RLF fails to survive the winter in mainland China and the early-summer adults are supposed to be overseas migrants. The Indochinese peninsula serves as the source of RLF population that migrates annually during April and May to the south of mainland China, where these moths reproduce three to four generations before subsequently expanding northward during June and July. Throughout late August to early September, RLF migrates back southward and leaves China by November [30]. RLF Migration is a key factor that induces the population abundance and infestation. This behavior is regulated not only by environmental conditions but also by its own physiological factors, with molecular modulation playing an important role in this process. The DGE profile indicated that several genes pertaining to RLF flight muscle cytoskeleton, including *flightin*, *Mlc1*, *Mlc2*, *Unc89*, *paramyosin*, *miniparamyosin*, *Tm2*, *TpnC*, *TpnI*, and *kettin*, were expressed at significantly higher levels in adults (Table S11). These genes directly determine the flight muscle structure and the flying ability of RLF. Muscle contractions in insects are under the simultaneous regulation by both myosin and actin [31], [32]. The troponin (Tpn) complex is composed of three closely interacting subunits, namely, Tpn C, Tpn T, and Tpn I, which promote contraction [33], [34]. The expression of *myosin* and *Tpn* plays a key role in RLF migration behavior. Additionally, several other proteins participate in the assembly and regulation of myofibrils, comprising paramyosin, actinin, myosin rod protein, flightin, and kettin [35]. We therefore propose that targeting the structural flight muscle genes may inhibit their expression, thereby degrading the muscles to control RLF.

The DGE profile indicated that the 12 genes pertinent to sex differences exhibited differential expression between the male and female moths; *Vg*, *VgR*, *YP4*, *Vg*, *HSP19.8*, *TSD*, *CarE7*, and *Tra2* genes demonstrated upregulated expression levels, whereas *PBP*, *OBP2*, *SFP*, and *CYP6CV1* genes showed downregulated expression levels in the female compared to the male moth. The results were further confirmed by qRT-PCR. We were surprised to find that *TSD* gene was differentially expressed between the male and female because such temperature-dependent sex determination (TSD) primarily exists in reptiles [36]. The small heat shock proteins are involved in the TSD molecular process [37], [38]. This result implies that RLF is likely to employ TSD in a similar manner as reptiles; however, this finding requires further investigation. Previous studies have shown that *PBP* and *OBP* genes are differentially expressed between the male and female adults [39], [40]; however, our DGE data have indicated that the corresponding mRNA expression levels are higher in RLF male moths than in female moths. In addition to detoxification functions, the three enzymes (GST, CarE7, and CYP6CV1) may also function as odorant-degrading enzymes (ODEs) that inactivate pheromones and other odorants [41], [42], [43]. In fact, there are numerous candidate genes with significant differential expression between males and females, thereby providing a valuable resource for further studies on sex determination and differentiation in RLF.

In summary, we have performed a comprehensive gene expression analysis in RLF. Although the biological functions of most RLF genes remain unclear, the transcriptome and DGE data provide worthwhile information for further research concerning development, sex differentiation, migratory flight, olfactory behavior, and insecticide resistance to assist in uncovering the underlying molecular mechanisms of this agricultural pest.

## Supporting Information

Figure S1 **Distribution of unigene lengths.**
(TIF)

Figure S2 **Distribution of total tag expression.** This figure shows the number and corresponding percentages of tags containing N, adapters, tags with copy number <2, clean tags, and raw tags. The numbers in parentheses show the quantity and percentage of each type of tag among the total raw tags.
(TIF)

Figure S3 **Distribution of copy number of total tags and distinct tags in each DGE library.** (A) Distribution of total clean tags. (B) Distribution of distinct clean tags. Total clean tags represent the sum of all clean tag numbers; distinct clean tags represent all types of clean tags. The number in square brackets indicates the range of copy number for a specific category of tags; the number in parentheses indicates the sum and percentage of corresponding tags among the total clean tags and distinct tags. For example, “[2], [5] (145385, 2.42%)” means that the tag number with 2 to 5 copies is 145,385, which accounts for 2.42% of total clean tags.
(TIF)

Table S1 **Primers used in qRT-PCR for confirmation of differentially expressed genes.**
(XLS)

Table S2 **The unigenes annotated by BLASTx against the NCBI Nr protein database.**
(XLS)

Table S3 **GO annotation of unigenes.**
(XLS)

Table S4 **COG annotation of unigenes.**
(XLS)

Table S5 **Sequence information of unigenes related to resistance.**
(XLS)

Table S6 **Sequence information of unigenes encoded odorant-binding proteins (OBPs).**
(XLS)

Table S7 **The 10 most upregulated and 10 most downregulated genes in each library of comparisons.**
(XLS)

Table S8 **Gene set enrichment analysis compared 3^rd^ instar larvae with eggs.**
(XLS)

Table S9 **Gene set enrichment analysis compared pupae with 3^rd^ instar larvae.**
(XLS)

Table S10 **Gene set enrichment analysis compared adults with pupae.**
(XLS)

Table S11 **Muscle cytoskeletal architecture-related genes.**
(XLS)

Table S12 **Genes related to sex differences.**
(XLS)

Table S13 **Top 10 specific expressed genes in each library comparison.**
(XLS)
