# Correction: Transcriptome and Gene Expression Analysis of the Rice Leaf Folder, *Cnaphalocrocis medinalis*

**DOI:** 10.1371/annotation/6e1416c2-0efe-47ef-968f-cf867ccf861c

**Published:** 2013-03-27

**Authors:** Shang-Wei Li, Hong Yang, Yue-Feng Liu, Qi-Rong Liao, Juan Du, Dao-Chao Jin

There was an error in the article title.

The correct title is: Transcriptome and Gene Expression Analysis of the Rice Leaf Folder, *Cnaphalocrocis medinalis*

The correct citation is: Li S-W, Yang H, Liu Y-F, Liao Q-R, Du J, et al. (2012) Transcriptome and Gene Expression Analysis of the Rice Leaf Folder, *Cnaphalocrocis medinalis*. PLoS ONE 7(11): e47401. doi:10.1371/journal.pone.0047401 

