# Supplementary figures and images for: Transcriptome and Gene Expression Analysis of the Rice Leaf Folder, Cnaphalocrosis medinalis
Source: PLoS One. 2012 Nov 19;7(11):e47401. doi: 10.1371/journal.pone.0047401 (PMC3501527; doi:10.1371/journal.pone.0047401)

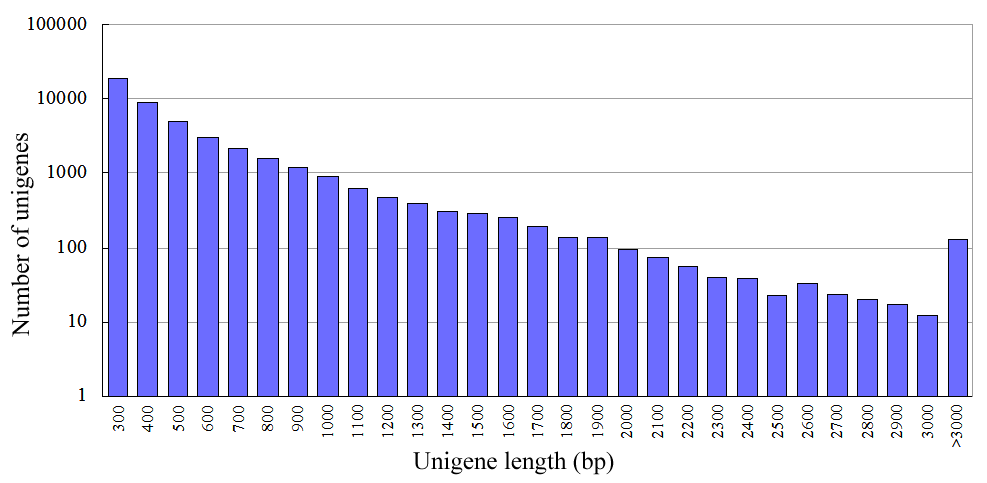

Supplement: Figure S1 — Distribution of unigene lengths. (TIF) [file pone.0047401.s001.tif]

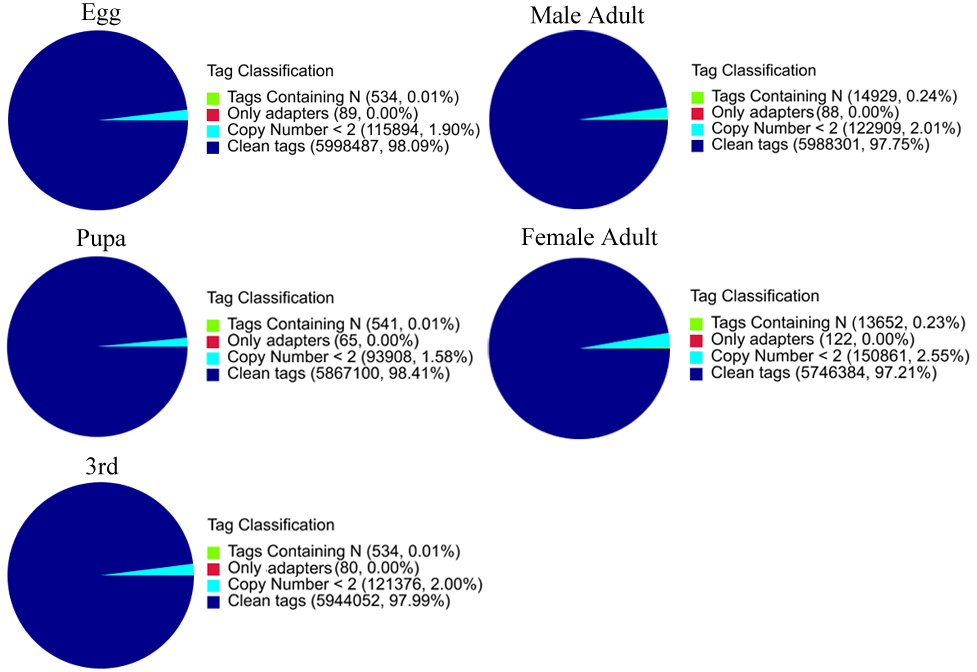

Supplement: Figure S2 — Distribution of total tag expression. This figure shows the number and corresponding percentages of tags containing N, adapters, tags with copy number <2, clean tags, and raw tags. The numbers in parentheses show the quantity and percentage of each type of tag among the total raw tags. (TIF) [file pone.0047401.s002.tif]

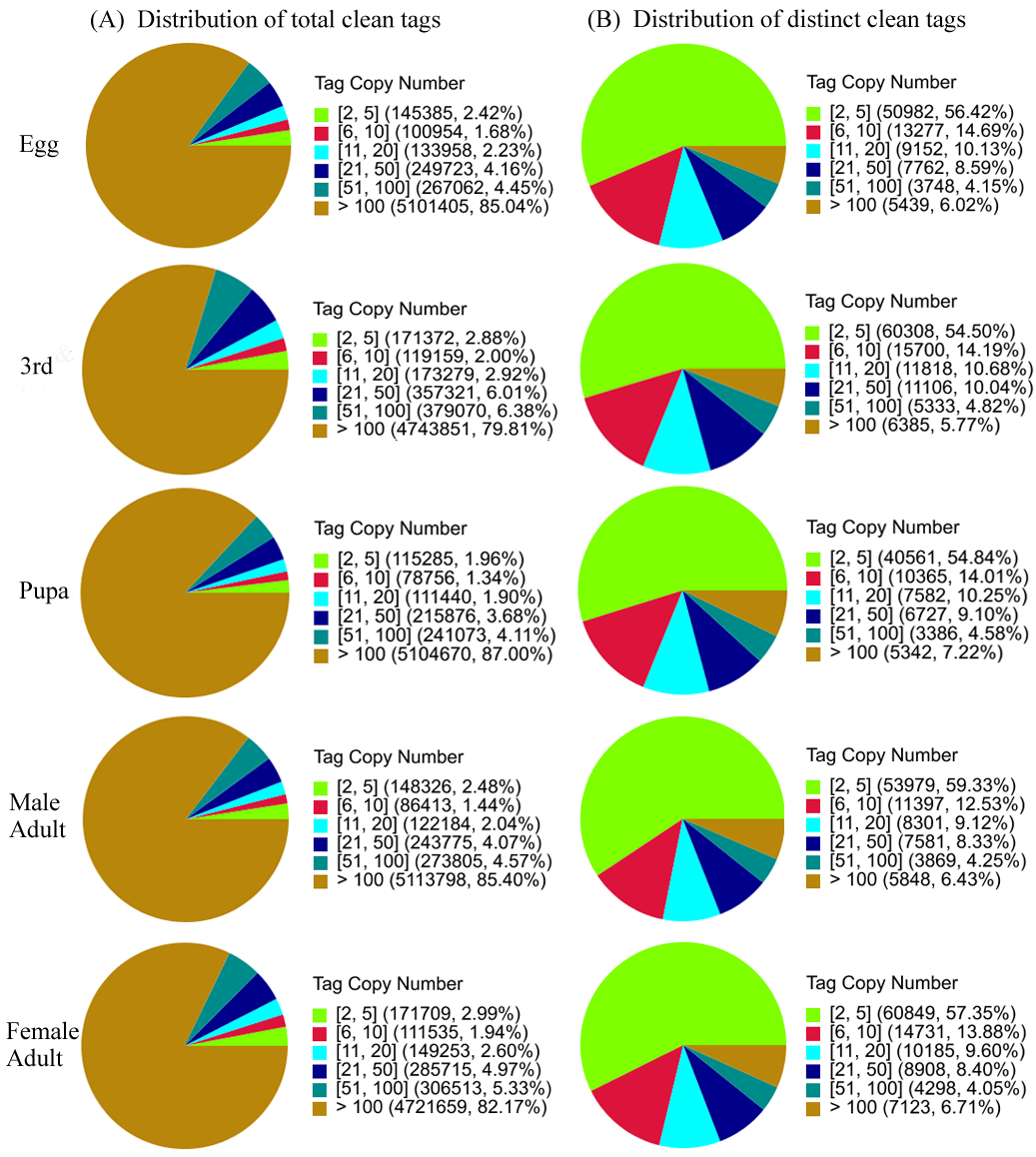

Supplement: Figure S3 — Distribution of copy number of total tags and distinct tags in each DGE library. (A) Distribution of total clean tags. (B) Distribution of distinct clean tags. Total clean tags represent the sum of all clean tag numbers; distinct clean tags represent all types of clean tags. The number in square brackets indicates the range of copy number for a specific category of tags; the number in parentheses indicates the sum and percentage of corresponding tags among the total clean tags and distinct tags. For example, “[2], [5] (145385, 2.42%)” means that the tag number with 2 to 5 copies is 145,385, which accounts for 2.42% of total clean tags. (TIF) [file pone.0047401.s003.tif]
